# Supplementary material for: Measurement of filtration efficiencies of healthcare and consumer materials using modified respirator fit tester setup
Source: PLoS One. 2020 Oct 13;15(10):e0240499. doi: 10.1371/journal.pone.0240499 (PMC7553287; doi:10.1371/journal.pone.0240499)
Supplement: S1 Table — Full list of and details on equipment and parts used in measurement set up. (DOCX) [file pone.0240499.s001.docx]

**S1 Table. Parts and equipment used in test setup**

| **Description** | **Manufacturer /Vendor** | **Part Number** | **Amount Needed** | **Approximate Cost** |
| --- | --- | --- | --- | --- |
| PortaCount Respirator Fit Tester | TSI | TSI 8038 | 1 |  |
| Particle Generator | TSI | TSI 8026 | 1 |  |
| Differential Pressure Gage | Dwyer | 1W479 | 1 | $83.00 |
| Volumetric Flow Meter | Brooks | 2KTW6 | 1 | $59.50 |
| Tri Clamp Bowl Reducer, 3 inch x 1.5 inch | Denord | DERNORD-301 | 2 | $65.98 |
| 1.5" Tri Clamp to 3/8" Hose Barbed Adapter | Denord | DERNORD-188 | 2 | $19.98 |
| Straight Adapter for 3/8" Hose ID, 1/8 NPTF Male | McMaster Carr | 5346K17 | 4 | $6.74 |
| 3" sanitary tri-clamp with 1 gasket | Denord | DERNORD-749 | 1 | $13.99 |
| 1.5" Sanitary tri-clamp w/ 1 gasket | Denord | DERNORD-624 | 2 | $11.95 |
| Tee Connector for 3/8" Hose ID | McMaster Carr | 91355K48 | 2 | $8.10 |
| Inline Tee, for 3/8" x 1/8" Tube ID | McMaster Carr | 5121K851 | 2 | $1.58 |
| Ball Check Valve, Polypropylene, 1/8", Barb | Grainger | 4DHX8 | 2 | $9.84 |
| Antistatic Silicone Rubber Tubing for Air and Water, Opaque Black, 3/8" ID, 1/2" OD, 5 ft | Mcmaster Carr | 1909T9 | 2.5 ft | $18.38 |
|  |  |  |  |  |
|  |  |  | **Total:** | **$299.04** |
