# Supplementary material for: Measurement of filtration efficiencies of healthcare and consumer materials using modified respirator fit tester setup
Source: PLoS One. 2020 Oct 13;15(10):e0240499. doi: 10.1371/journal.pone.0240499 (PMC7553287; doi:10.1371/journal.pone.0240499)
Supplement: S2 Table — Complete list of and details of materials tested, and filtration efficiency and pressure drop results. (DOCX) [file pone.0240499.s002.docx]

**S2 Table. Material details and complete results**

| **Material** | **Material Details** | **Flowrate (Lpm)** | **FE (%)** | **FE (SD)** | **ΔP** |
| --- | --- | --- | --- | --- | --- |
| **N95 1870** | 3M Aura model 1870+ | **10** | 99.96 | ~ | 2.54 |
|  |  | **25** | 99.43 | 0.18 | 15.24 |
|  |  | **40** | 99.25 | ~ | 6.35 |
|  |  | **60** | 98.45 | ~ | 22.86 |
| **N95 1860** | 3M model 1960 | **25** | 98.89 | ~ | 12.7 |
| **HY8510** | Tronex | **25** | 92.95 | 0.36 | 12.7 |
| **H500** | 2 layers of NWPP fused peripherally, Halyard, H500 | **25** | 86.35 | 1.67 | 22.86 |
| **H100** | 2 layers of NWPP fused peripherally, Halyard, H100 | **25** | 79.83 | 2.55 | 10.16 |
| **ABD Pad** | Kendall Curity Abdominal Pad, Coviden, 9190A | **25** | 75.47 | 1.63 | 10.16 |
| **Surgical Mask** | Hospital standard issue; model number not available | **25** | 74.36 | 4.54 | 6.35 |
| **Sterilization**  **Box Filter** | Steritite filter, Case Medical SCF02 | **25** | 66.83 | 5.94 | 12.7 |
| **Pediatric Drape** | Cardinal Health, 29492 | **25** | 50.22 | 4.52 | 12.7 |
| **Bair Cover** | 3M Bair Hugger Warming Gown, 81002 | **25** | 48.94 | ~ | 10.16 |
| **Surgical Gown** | Cardinal Health Non-reinforced, AAMI Level 3, Size XXL, 9545 | **25** | 42.03 | 7.58 | 12.7 |
| **Chux** | Cardinal Health Wings Quilted Premium Comfort Underpad, P3036C | **25** | 33.57 | ~ | 7.62 |
| **Shoe Cover** | Cardinal Health SMS covers, 4852 | **25** | 31.62 | ~ | 6.35 |
| **Mayo Stand**  **Cover** | Cardinal Health Convertors, 8337 | **25** | 12.67 | ~ | 10.16 |
| **Vacuum Bag** | 3M Filtrete Eureka MM, paper removed | **25** | 82.54 | 2.06 | 12.7 |
| **HVAC Filter** | Honeywell HW1625-13R; Merv 13 | **25** | 52.15 | 6.94 | 0 |
| **Smart-Fab** | Black, model number 1U67482420 | **25** | 48.59 | 3.51 | 12.7 |
| **Interfacing** | Pellon 915 100% polypropylene | **25** | 42.13 | 3.43 | 2.54 |
| **Lawn Fabric** | Non-woven polypropylene landscaping fabric, 3oz thickness | **25** | 39.07 | 2.18 | 10.16 |
| **Shopping Bag** | Non-woven polypropylene, issued by local public library | **25** | 37.88 | 1.65 | 10.16 |
| **Paper Towel** | Bounty Advanced 2-ply | **25** | 36.64 | 8.16 | 7.62 |
| **Pillowcase** | Allersoft dust mite & bed bug barrier | **25** | 36.57 | 3.73 | 12.7 |
| **T shirt** | Gildan 100% Heavy Cotton | **25** | 35.77 | 5.28 | 12.7 |
| **Cotton** | Novelty cotton fabric 100% | **25** | 28.20 | 2.63 | 6.35 |
| **Coffee Filter** | Chemex brand | **25** | 17.31 | ~ | 12.7 |
